# Supplementary material for: Integrin-Specific Signaling Drives ER Stress-Dependent Atherogenic Endothelial Activation
Source: bioRxiv. 2025 Jun 1:2025.05.31.654582. Preprint. [Version 1] doi: 10.1101/2025.05.31.654582 (PMC12154597; doi:10.1101/2025.05.31.654582)
Supplement: 1 — Supplementary Figure 1: Integrin activation drives ER stress. (A-C) MLECs isolated from Talin WT and Talin1 L325R, plated on fibronectin, treated with the indicated concentration of tunicamycin (10 μg/ml, 24 h). and ER stress markers (P-eIF2α and XBP1s) were assessed by Western blotting. P values were determined by two-way ANOVA with Tukey’s multiple comparisons test. Data are presented as mean ± SEM. Each point represents one independent experiment. Supplementary Figure 2: Fibronectin selectively amplifies ER stress in endothelial cells independent of global translation and oxidative stress. (A-B) HAECs plated on fibronectin or basement membrane were treated with OxLDL (100 μg, 18h) or (C-D) subjected to OSS (±5 dynes/cm2 with 1 dyne/cm2 forward flow, 18h) and then treated with puromycin (2μg/ml, 30min). Puromycin was assessed by Western blotting. (E) Representative images of HAECs plated on either Matrigel or fibronectin, treated with OxLDL (100 μg, 24 h) or (F) subjected to OSS (±5 dynes/cm2 with 1 dyne/cm2 forward flow, 18h), and stained for Lamin and TPE-MI. (G) HAECs plated on fibronectin or basement membrane were subjected to OSS (±5 dynes/cm2 with 1 dyne/cm2 forward flow, 18h) and then treated with DHE (2μM, 30min). Superoxide was measured. (H) Representative Western blot of HAECs pretreated with TEMPOL (500uM, 24 h) and then treated with either controls or activating α5β1 or αvβ3 CHAMPs (4uM, 24 h). ER stress markers (XBP1s and P-eIF2α) were assessed by Western blotting. P values were determined by two-way ANOVA with Tukey’s multiple comparisons test. Data are presented as mean ± SEM. Each point represents one independent experiment. [file NIHPP2025.05.31.654582V1-supplement-1.pdf]

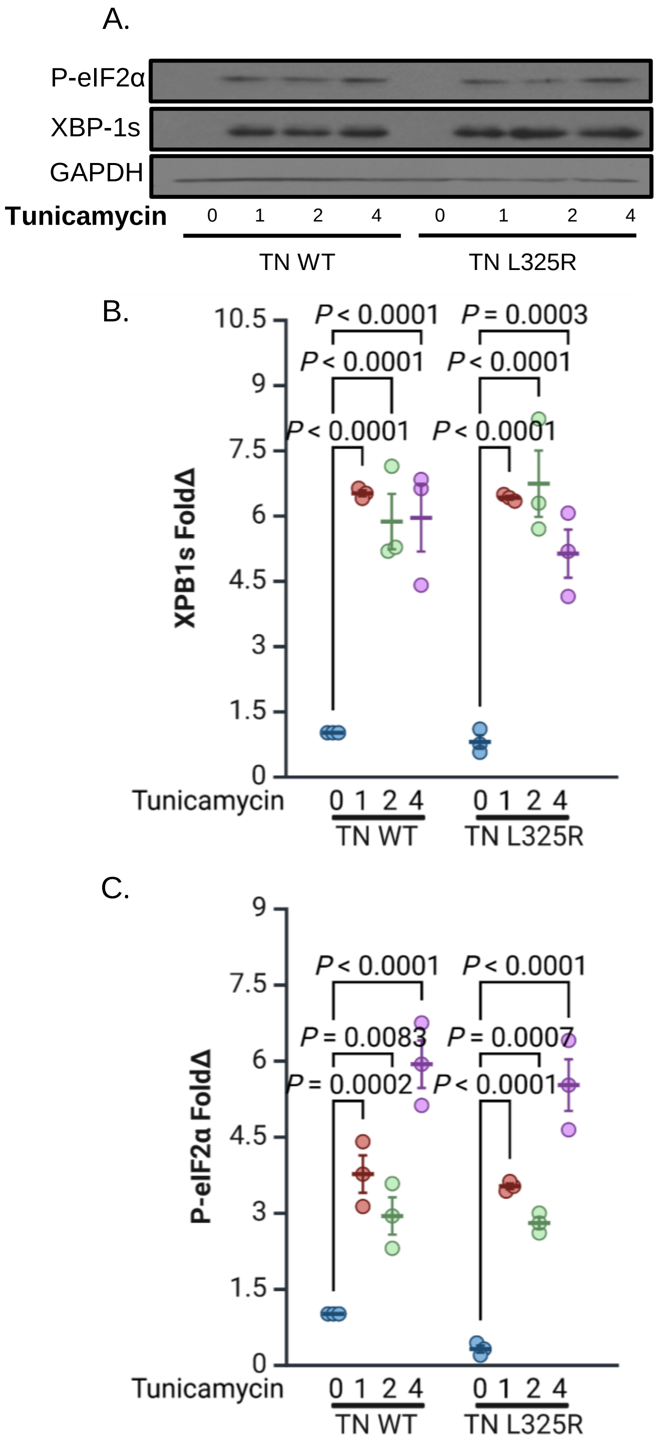

Supp Figure 1

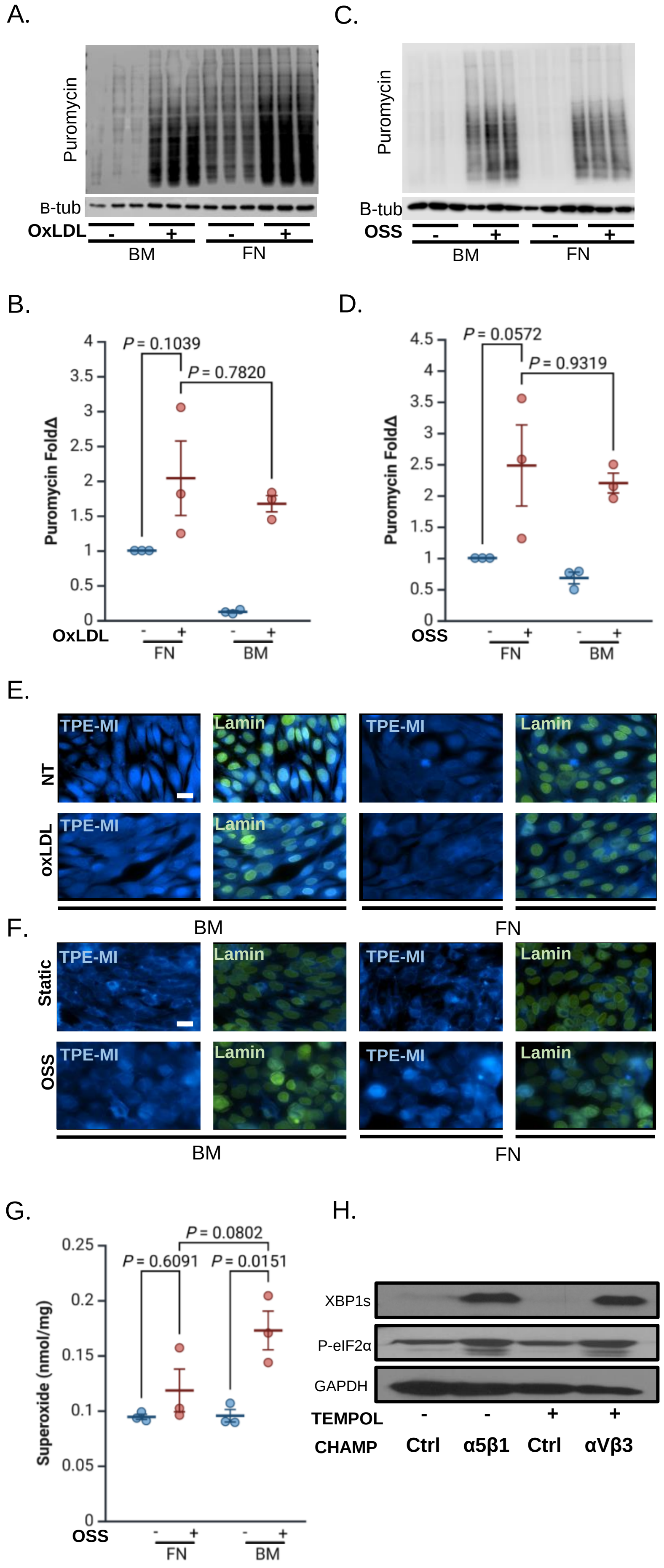

Supp Figure 2

Target	Source	Catalog Number	Application	Working Concentration
XBP-1s	Cell Signaling	# 47134	IHC/ICC	1:200 (IHC)<br>1:500 (ICC)
XBP-1s	Biolegend	#647501	WB	1:400 (WB)
P-eIF2α	Cell Signaling	3398	IHC	0.1 μg/mL
ATF4	Cell Signaling	11815	WB	1:1000
BIP	Abcam	ab21685	WB/IHC	1:1000 (WB)<br>1:200 (IHC)
NRF2	Santa Cruz	SC-722	WB	1:1000
GAPDH	Cell Signaling	#2118	WB	1:5000
α5 integrin	Abcam	ab150361	WB	1:1000
B3 integrin	Abcam	ab44-878	WB	1:1000
CD31	Santa Cruz	Sc-1506	IHC	4 μg/mL
VCAM-1	Abcam	Ab134047	WB	1:1000
ICAM-1	Santa Cruz	sc-138	WB	1:200
P-JNK (Ser473)	Cell Signaling	#4668	WB	1:1000
JNK	Cell Signaling	#9252	WB	1:1000
Phospho-NF-κB (Ser536, p65 subunit)	Cell Signaling	#3033	WB	1:1000
NF-κB (p65 subunit)	Cell Signaling	#4764	WB	1:1000
Puromycin	Sigma	#MABE343	WB	1:25000
C-JUN	Cell Signaling	#9165	IHC	1:500

Supp Table 1
